# SFI, a sex hormone binding globulin based nomogram for predicting non-alcoholic fatty liver disease in the Chinese population

**DOI:** 10.3389/fendo.2023.1176019

**Published:** 2023-06-06

**Authors:** Xiaomin Hua, Heping Zhang, Wenru Yang, Guotao Liu, Suhua Zhang, Yingcui Wang

**Affiliations:** ^1^Department of Health Care, Qilu Hospital (Qingdao), Cheeloo College of Medicine, Shandong University, Shandong, China; ^2^Department of Cardiac Surgery, the Affiliated Hospital of Qingdao University, Shandong, China; ^3^Department of Cardiology, Qilu Hospital (Qingdao), Cheeloo College of Medicine, Shandong University, Shandong, China

**Keywords:** non-alcoholic fatty liver disease, predictive model, sex hormone binding globulin, nomogram, SFI

## Abstract

**Background:**

The purpose of this study is to establish a novel nomogram model for accurate detection of non-alcoholic fatty liver disease (NAFLD) in the Chinese population based on sex hormone binding globulin (SHBG) and other routine laboratory tests.

**Methods:**

A total of 1417 participants (1003 testing and 414 validations) were enrolled into the study. Risk factors independently associated with NAFLD were identified and incorporated in the new nomogram, SFI. The performance of nomogram was assessed by analysis of receiver operating characteristic (ROC) curve, calibration curve, and decision curve.

**Results:**

We formulated a new nomogram incorporating four independent factors: SHBG, body mass index (BMI), ALT/AST, and triglycerides (TG). The nomogram achieved good indexes of area under ROC 0.898 (95% confidence interval 0.865–0.926) in predicting NAFLD, which was significantly superior to previously reported models of FLI, HSI, LFS, and LAP. The calibration curve and decision curve demonstrated high performance and clinical utility of the nomogram in predicting NAFLD.

**Conclusion:**

The nomogram SFI has high performance in predicting NAFLD in Chinese population and may be used as a cost-effective screening model to assess NAFLD in the general population.

## Introduction

Due to the improvement of living standards and unhealthy lifestyles, the prevalence of Nonalcoholic fatty liver disease (NAFLD) is increasing in western and Asian countries. In China, the prevalence of fatty liver during the last decade has been estimated to be 15-40% (1). Because of the close relationship with metabolic syndrome (Mets), NAFLD occurs in about 40-60% of type 2 diabetes patients and 80% of obese patients (2–4). As the most common cause of chronic liver disease, NAFLD ranges from simple steatosis to steatohepatitis, advanced fibrosis, cirrhosis, and even hepatocellular carcinoma (5, 6), leading to an increase in all-cause mortality and placing a huge burden on medical systems worldwide (7, 8).

Most individuals with NAFLD do not have specific symptoms, especially at the early stage, which limits prevention and detection of NAFLD (9).

Therefore, early detection of NAFLD is essential to identify those with potentially asymptomatic progressive fatty liver disease. Traditionally, liver biopsy remains the golden standard for NAFLD diagnosis, but it is considered impractical because of its invasiveness, costly, and the prone to sampling bias (10, 11). Imaging examination of ultrasonography (US), computed tomography (CT), and magnetic resonance imaging (MRI) are mostly used as alternatives to diagnose NAFLD, but they are also not cost-effective, and not suitable for large-scale population screening. Therefore, it is necessary to develop an effective clinical prediction model, which can be widely adapted in different medical centers for an early detection of NAFLD.

In previous studies, clinicians have been trying to develop simple non-invasive scoring systems for predicting NAFLD. The fatty liver index (FLI), which incorporates body mass index (BMI), waist circumstance (WC), gamma-glutamyl-transferase (GGT), and triglycerides (TG), has been validated to have high accuracy in detecting hepatic steatosis (12, 13). Other algorithms, such as the NAFLD Liver Fat Score (LFS) (14), the Hepatic Steatosis Index (HSI) (15), and the Lipid Accumulation Product (LAP) (16), etc. are also useful for screening NAFLD. We noted that most of the algorithms were based on routine anthropometric and metabolic parameters, which showed poorer agreement with imaging in the assessment of steatosis, and are not widely validated in general Chinese populations.

Sex hormone binding globulin (SHBG) is a glycoprotein synthesized in the liver and has been found to be associated with metabolic disorders (17, 18). Emerging evidence from epidemiologic studies suggest that, SHBG is increasingly recognized as a hepatokine that involved in the occurrence and development of NAFLD (19, 20).

Therefore, in the current study, we established a nomogram, which not only incorporating routine clinical and laboratory tests, but also innovatively included SHBG as an important indicator to diagnose NAFLD. We compared the diagnostic accuracy of NAFLD-screening models and carried out the calibration curves and decision curve analysis (DCA) to evaluate the performance and clinical utility of this nomogram.

## Methods

### Study participants

**Figure 1:**
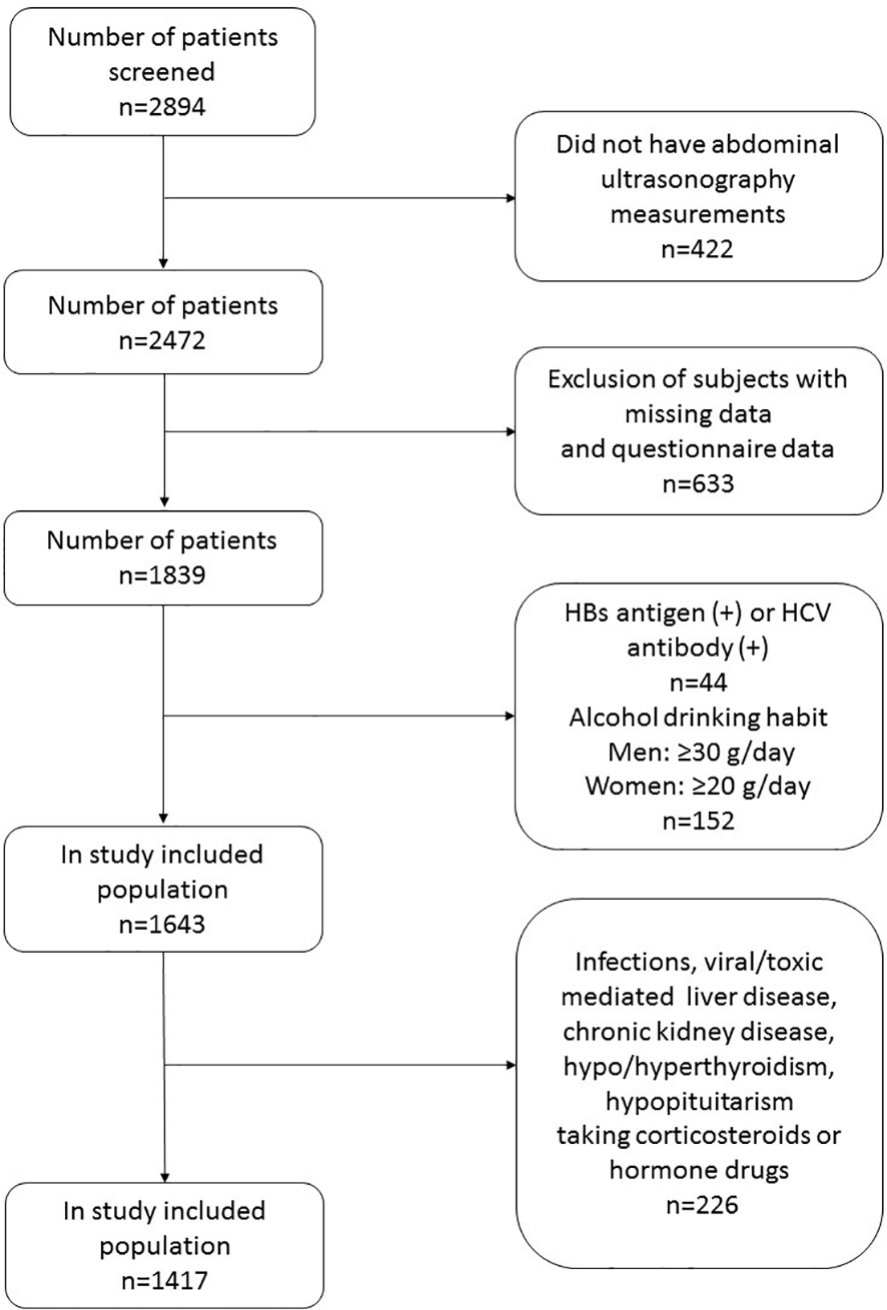
The study flow chart.

A total of 2894 adult patients who visited the department of Health Care, Qilu Hospital (Qingdao), Shandong University between January 2015 and December 2019 were sequentially enrolled in this registry. Information on smoking habit, alcohol drinking habit, and personal medical history were obtained from the subjects. A flow chart of the study participants is shown in **Figure 1**. Exclusion criteria were subjects with hepatitis B surface antigen positive, hepatitis C antibody positive and excessive alcohol consumption (alcohol equivalent: ≥30 g/day for men, ≥20 g/day for women), and subjects who lacked data on abdominal ultrasonography, sex hormones, and fatty liver assessment formula components. In order to study the role of SHBG in fatty liver, we also excluded subjects with acute/chronic infection, viral/toxic mediated liver diseases, chronic kidney disease, hypo/hyperthyroidism, hypopituitarism, and history of taking corticosteroids or sex hormone replacement.

After prespecified exclusion, a total of 1417 Chinese subjects (men/women: 869/548; mean age: 62.12 ± 13.9 years) were finally recruited in the present study. The original dataset was randomly divided into training dataset (1003 cases) for developing prediction model and validation dataset (414 cases) for model validation according to 7:3 ratio (21). Model training was performed numerous times.

The study was in accordance with the principles outlined in the Declaration of Helsinki and was approved by the Ethics Committee of Qilu Hospital (Qingdao), Shandong University. Written informed consent was obtained from all participants.

### Clinical and laboratory measurements

Height and body weight were measured to the nearest millimeter and kilogram. BMI was calculated as weight in kilograms divided by height in meters squared. WC was measured at an intermediate level between the lowest rib and the iliac crest. Blood pressure was measured using standard method as in a seated position after rest for 15 more minutes.

After fasting for 12 hours, venous blood samples were drawn from all subjects before 10:00 a.m. All biochemical assays were performed in the same laboratory using standard methods. Laboratory tests included: alanine aminotransferase (ALT), aspartate aminotransferase (AST), GGT, serum fasting plasma glucose (FPG), haemoglobin A1c (HbA1c), total cholesterol (TC), TG, high density lipoprotein cholesterol (HDL-C), low density lipoprotein cholesterol (LDL-C), fasting insulin (FINS), fasting C-peptide(FCP), and SHBG. The following equation was used for the homeostasis model assessment of insulin resistance: HOMA-IR= FPG(mmol/L)*FINS(mU/L)/22 (16).

### US assessments for NAFLD

Two well-experienced ultrasonographers, who were blinded to the clinical data, performed the abdominal US examination to diagnose NAFLD. The diagnostic criteria for fatty liver by ultrasonography included: increased echogenicity in the hepatic parenchyma than in the kidney, blurred intrahepatic vessel structure, and abnormal visualization of diaphragm and posterior right hepatic lobe (22).

### Definition of variables

The diagnosis of MetS was considered based on the International Diabetes criteria (23): (1) abdominal obesity (waist circumference ≥90 cm for Asian men or ≥80 cm for Asian women); (2) at least two of the following four components: 1) triglycerides ≥150 mg/dl (1.7 mmol/L), or specific treatment for this lipid abnormality; 2) HDL cholesterol<40 mg/dl (1.03 mmol/L) for men or<50 mg/dl (1.29 mmol/L) for women, or specific treatment for this lipid abnormality; 3) systolic blood pressure (SBP)/diastolic blood pressure (DBP) ≥130/85 mmHg or receiving drug treatment; and 4) fasting plasma glucose ≥100 mg/dl (5.6 mmol/L), or previously diagnosed T2D or receiving treatment.

Diagnosis of diabetes mellitus was based on the guideline of the American Diabetes Association in 2014: FPG ≥7.0mmol/L or HbA1c≥6.5%, or a previous diagnosis made by a healthcare professional.

### NAFLD prediction algorithms


$$\begin{array}{c} FLI=\;(e^{0.953\times loge\left(triglycerides\right)\;+\;0.139\times BMI+\;0.718\times loge\left(GGT\right)\;+\;0.053}\\ {}^{\times WC-\;15.745})\;/\;(1\;+\;e^{0.953\times loge\left(triglycerides\right)\;+\;0.139\times BMI+}\\ {}^{0.718\times loge\left(GGT\right)\;+\;0.053\times WC-\;15.745})\;\times 100 \end{array}$$

$$\begin{array}{c} LFS=-2.89+1.18\times MetS(yes=1/no=0)+0.45\times type\;2\;diabetes\\ \;(yes=2/no=0)+0.15\times FINS(mU/L)+0.04\times AST-0.94\\ \times AST/ALT. \end{array}$$

$$\begin{array}{c} LAP=(waist\;circumference-65)\times (triglycerides)\;in\;men\;and\\ \;(waist\;circumference-58)\times (triglycerides)\;in\;women. \end{array}$$

$$\begin{array}{c} HSI=8\times (ALT/AST\;ratio)+\\ \;BMI(+2,\;if\;female;\;+2,\;if\;diabetes\;mellitus). \end{array}$$

### Statistical analysis

Continuous and categorical variables were expressed as means ± standard deviation (SD) and percentage (%), respectively. Comparison of continuous variables was done with Student’s t-test or Mann-Whitney’s *U*-test, and categorical parameters were compared using the chi-square test. Variables that were statistically significant by univariate analyses were added to a multiple logistic regression model to identify independent predictors for the presence of NAFLD after adjusting for age and sex. Based on the result of multiple logistic regression analysis, we formulated a nomogram by proportionally converting regression coefficient to a 0- to 100-point scale. The variable with the highest β coefficient was assigned 100 points. Accordingly, the points across the respective variables were added to obtain total points, which were converted into prediction probabilities. The area under the receiver-operating characteristic (AUROC) as with sensitivity and specificity were constructed and compared to evaluate the predictive power of indices for diagnosing NAFLD. Effective cutoff values were obtained by calculating the Youden’s index from ROC curves (24). The predictive performance of nomogram was measured by calibration using 1000 bootstrap samples to reduce the over fitting bias. By quantifying the probability of net benefits at a threshold from 0.0 to 1.0, we conducted DCA curve to evaluate the clinical utility of SFI. All analyses were performed using SPSS version 22.0 (Chicago, IL, USA) and R version 4.0.2 (College Station, Texas, USA). *P* value<0.05 was considered statistically significant.

## Results

### Baseline characteristics of the participants

1003 patients were enrolled into the training dataset and 414 patients were enrolled into the validation dataset. Through ultrasonography examination, NAFLD detection rate was 423 (42.22%) in the training dataset and 183 (44.2%) in the validation dataset. Male subjects had a significantly higher rate of NAFLD detection rate than female in both datasets (*P*< 0.001). A total of 832 (58.7%) patients were diagnosed with MetS. The incidence of MetS in NAFLD group was higher than that in non-NAFLD group (*P*< 0.001). The demographic and laboratory features of all subjects are provided in **Table 1**. According to the univariate analysis, patients with NAFLD had higher BMI, waist circumference, and DBP, higher levels of ALT, AST, ALT/AST ratio, GGT, TC, TG, FBG, HbA1c, FINS, FCP, and HOMA-IR, but significantly lower HDL levels, in comparison with patients without NAFLD in training cohort (*P*< 0.05). In addition, SHBG levels were significantly lower in patients with NAFLD than that without (25.78 ± 11.01 nmol/L vs. 45.71 ± 18.1 nmol/L, *P*< 0.001). The clinical features in the validation dataset were comparable to that in the training dataset (see **Supplement Table S1**).

**Table 1:** Baseline characteristics of patients with and without NAFLD in the training dataset.

	Total (n = 1003)	non-NAFLD (n = 580)	NAFLD (n = 423)	*P* value
Sex (Male/Female)	612/391	324/256	288/135	< 0.001
Age (years)	62.53 ± 13.83	66.17 ± 13.34	57.55 ± 12.91	< 0.001
Mets (%)	587 (58.5)	274 (47.2)	313 (74.0)	< 0.001
Smoking (%)	224 (22.3)	112 (19.3)	112 (26.5)	0.005
Drinking (%≥1/w)	99 (9.9)	40 (6.9)	59 (13.9)	0.000
SBP (mmHg)	134.14 ± 18.79	133.62 ± 19.66	134.84 ± 17.53	0.311
DBP (mmHg)	76.54 ± 12.49	7402 ± 12.29	79.99 ± 11.94	< 0.001
BMI (kg/m^2^)	24.78 ± 3.33	23.52 ± 2.97	26.50 ± 3.01	< 0.001
WC (cm)	91.00 ± 9.80	87.82 ± 8.94	95.37 ± 9.24	< 0.001
ALT (U/L) *	24.20 ± 16.84	19.61 ± 11.56	30.48 ± 20.53	< 0.001
AST (U/L)*	21.09 ± 10.73	19.291 ± 6.75	23.54 ± 14.16	< 0.001
ALT/AST	1.13 ± 0.43	1.01 ± 0.36	1.30 ± 0.45	< 0.001
GGT (U/L)*	31.88 ± 28.77	24.39 ± 19.61	42.15 ± 35.43	< 0.001
TC (mmol/L)	4.40 ± 1.10	4.28 ± 1.03	4.44 ± 1.13	0.030
TG (mmol/L)*	1.79 ± 1.49	1.30 ± 0.85	2.27 ± 1.53	< 0.001
HDL-C (mmol/L)	1.13 ± 0.46	1.21 ± 0.42	1.02 ± 0.47	< 0.001
LDL-C (mmol/L)	2.32 ± 0.82	2.30 ± 0.78	2.29 ± 0.81	0.940
FPG (mmol/L)	7.49 ± 3.50	7.02 ± 3.36	8.13 ± 3.57	< 0.001
HbA1c (%)	8.42 ± 2.40	8.20 ± 2.52	8.72 ± 2.18	0.001
FINS (mU/L)*	11.21 ± 15.11	10.87 ± 18.27	11.63 ± 10.04	< 0.001
FCP (pmol/L)*	758.00 ± 436.74	639.61 ± 403.17	903.31 ± 432.94	< 0.001
HOMA-IR	3.68 ± 1.70	3.23 ± 1.37	4.22 ± 1.89	< 0.001
SHBG (nmol/L)	37.30 ± 18.71	45.71 ± 18.71	25.78 ± 11.01	< 0.001

Continuous data were performed as the Student’s T-test or Mann-Whitney’s U-test* and presented as means **±** SD. Categorical variables are presented as proportion and performed as χ^2^**-**test.

### Derivation of a new index for NAFLD

**Figure 2:**
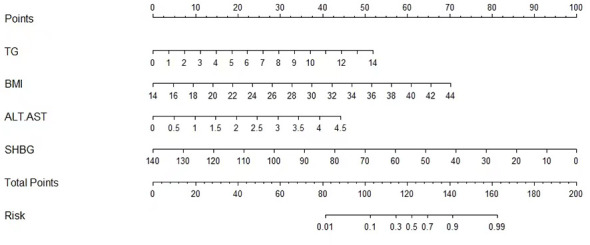
Nomogram for predicting NAFLD.

**Table 2** presents the results of logistic regression analysis among variables that related to NAFLD. Because of the significant interactions and multicollinearities in the same kind of variables, we selectively incorporated the variables with the highest odds ratios (OR) into the further multiple logistic regression. After adjusted for age and sex, four independent predictors, BMI, ALT/AST, TG, and SHBG, with the best performance incorporated into our model and presented as the nomogram (**Figure 2**).

**Table 2:** Univariate and multivariate analyses for the prediction of NAFLD.

		Univariate logistic regression analysis			Multiple logistic regression model		
Variable	AUC	β	OR (95%CI)	*P* Value	β	OR (95%CI)	*P* Value
Age (years)	0.325	-0.047	0.954 (0.946-0.962)	< 0.001	-0.009	0.991 (0.9780-1.005)	0.216
Sex	0.551	0.574	1.775 (1.423-2.214)	< 0.001	-0.260	0.771 (0.550-1.081)	0.132
BMI (kg/m^2^)	0.755	0.336	1.399 (1.338-1.463)	< 0.001	0.276	1.318 (1.242-1.400)	< 0.001
WC (cm)	0.709	0.091	1.096 (1.081-1.111)	< 0.001			
SBP (mmHg)	0.511	0.004	1.004 (0.999-1.010)	0.150			
DBP (mmHg)	0.630	0.039	1.039 (1.030-1.049)	< 0.001	0.010	1.010 (0.997-1.024)	0.131
ALT (U/L)	0.723	0.058	1.060 (1.050-1.071)	< 0.001			
AST (U/L)	0.611	0.047	1.048 (1.034-1.061)	< 0.001			
ALT/AST	0.706	1.966	7.142 (5.253-9.709)	< 0.001	1.237	3.445 (2.383-5.197)	< 0.001
GGT (U/L)	0.766	0.036	1.037 (1.029-1.044)	< 0.001			
FBG (mmol/L)	0.585	0.087	1.090 (1.058-1.124)	< 0.001	-0.009	0.991 (0.933-1.052)	0.760
HbA1c	0.535	0.073	1.076 (1.029-1.125)	0.001			
FINS (mU/L)	0.646	-0.005	0.995 (0.987-1.003)	0.224			
FCP (pmol/L)	0.700	0.002	1.002 (1.001-1.002)	< 0.001			
HOMA-IR	0.700	0.452	1.572 (1.439-1.718)	< 0.001	0.102	0.989 (0.977-1.001)	0.084
TG (mmol/L)	0.752	0.785	2.191 (1.924-2.496)	< 0.001	0.157	1.170 (1.008-1.357)	0.038
HDL-C (mmol/L)	0.676	-1.149	0.317 (0.233-0.431)	< 0.001			
SHBG (nmol/L)	0.840	-0.106	0.900 (0.889-0.910)	< 0.001	-0.088	0.916 (0.902-0.930)	< 0.001

As a result, we derived an equation, which we named the $\begin{array}{l} SFI=(e^{0.420\;\times \;\mathrm{loge}\;(triglycerides)\;+\;0.265\;\times \;BMI+\;1.112\;\times ALT/AST-0.081\times SHBG-0.0624})/\\ (1+e^{0.420\;\times \;\mathrm{loge}\;(triglycerides)\;+\;0.265\;\times \;BMI+\;1.112\;\times ALT/AST-0.081\times SHBG-0.0624})\;\times 100 \end{array}$

### Accuracy of NAFLD diagnosis

**Figure 3:**
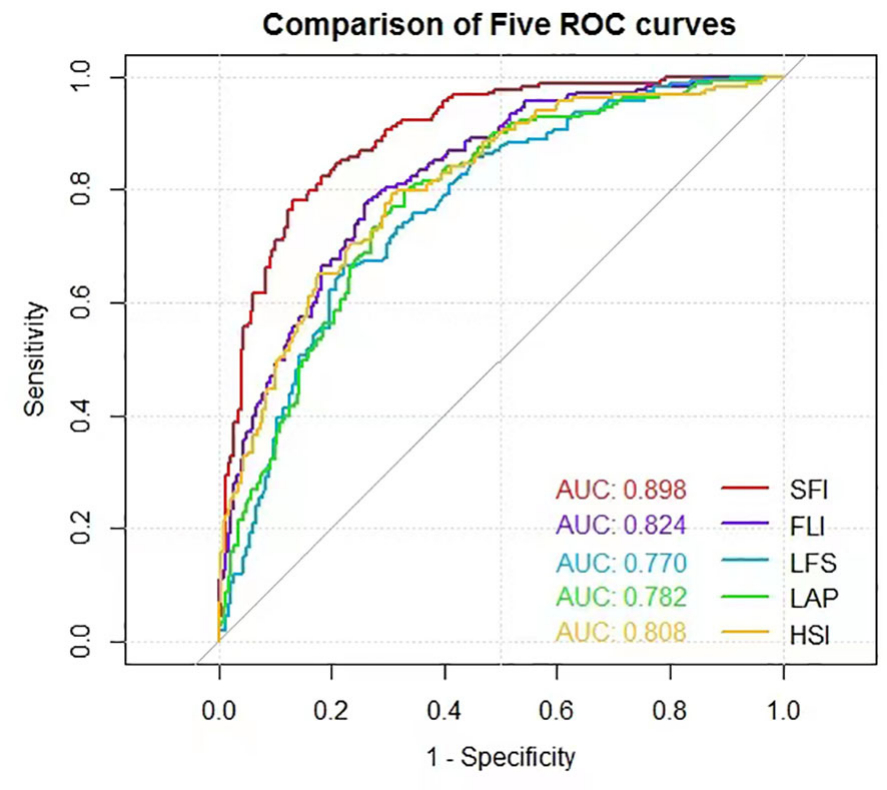
ROC curves for predicting NAFLD in validation dataset.

In the validation dataset, the AUROCs were calculated to compare the diagnosis accuracy of the newly built nomogram and other four models in predicting NAFLD. The nomogram SFI had the highest AUROC (0.898, 95% CI 0.865–0.926, *P*< 0.001) for predicting NAFLD compared with AUROCs of the FLI (0.824, 95% CI 0.784–0.860, *P*< 0.001), the LFS (0.770, 95% CI 0.722–0.814, *P*< 0.001), the LAP (0.782, 95% CI 0.739–0.821, p<0.001), and the HSI (0.808, 95% CI 0.766–0.845, *P*< 0.001). Sensitivity and specificity of SFI were 78.1% and 87.1%, respectively, and the cut-off was 55.246 (**Figure 3**). Details of the performance are shown in **Table 3**.

**Table 3:** Accuracy of the prediction score of SFI, FLI, LFS, LAP, and HSI for the prediction of NAFLD.

Models	AUC (95%CI)	*P* values	Cut-offs	Sensitivity (%)	Specificity (%)
SFI	0.898(0.865-0.926)	<0.001	55.246	78.1	87.0
FLI	0.824(0.784-0.860)	<0.001	40.09	78.1	73.6
LFS	0.770(0.722-0.814)	<0.001	0.350	66.7	77.7
LAP	0.782(0.739-0.821)	<0.001	39.075	79.8	67.1
HSI	0.808(0.766-0.845)	<0.001	36.045	79.2	69.3

Additionally, when we restricted the subjects to BMI<23kg/m^2^, the nomogram even shows a better result of AUROC (0.940, 95%CI 0.899-0.982), suggesting that this model is also applicable to the non-obese NAFLD population (**Supplement Figure S1**).

### Performance and clinical utility of the nomogram

**Figure 4:**
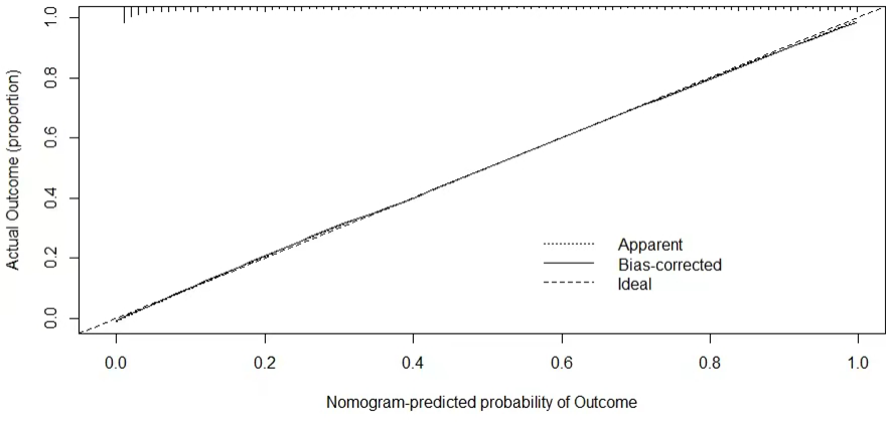
Validity of the predictive performance of nomogram SFI in estimating the presence of NAFLD.

The calibration curve was conducted to estimate the validity of the predictive performance of the nomogram in the validation dataset (**Figure 4**). A closer fit of the solid line to the diagonal dotted line represents a better prediction. The calibration curve of SFI demonstrated a perfect prediction model for predicting NAFLD with optimal agreement with actual US examination. For the participants, the C-index for the prediction nomogram was 0.888 (95% CI: 0.868–0.907).

**Figure 5:**
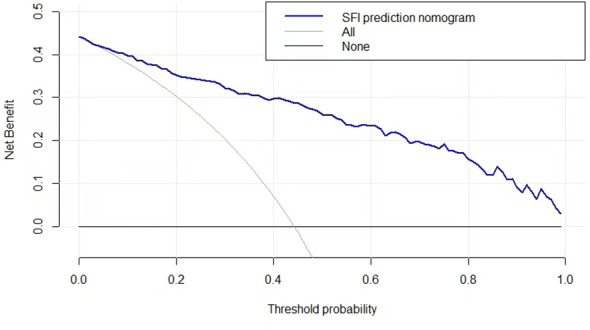
The decision curve to assess the clinical utility of the nomogram.

DCA was conducted to evaluate the clinical utility of the SFI. When the decision curve is far from the two extreme curves, the clinical decision net of the model can benefit high. According to the current decision curve, the nomogram SFI for predicting NAFLD demonstrated high net benefit in the validation dataset (**Figure 5**).

## Discussion

With the increasing prevalence of NAFLD and the concomitant hepatic and extrahepatic consequences, it has become an urgent public health problem. Developing simple and cost-effective screening methods has become extremely important. In the present study, we devised a novel nomogram SFI incorporating SHBG and routine parameters of BMI, ALT/AST, and TG, for identifying the presence of NAFLD. The high performance and benefit of the nomogram for detecting NAFLD has been confirmed.

In the past decades, several studies have attempted to devise noninvasive methods that can predict the presence of NAFLD. FLI, HSI, LAP, and LFS are simple and non-invasive models that combine biochemical tests and anthropometric data to predict fatty liver in people of different races and countries (12, 14–16). The parameters of high-frequency application mainly include sex, liver enzymes (GGT, ALT, AST), and components of MetS (BMI, WC, blood pressure, FPG, TG). These parameters above can usually be monitored during the annual physical examination or when people visiting the clinic. Thus, we have taken into account these factors and selected the mostly influenced ones. In addition, we added a new index SHBG as one of the important predictors to the model.

SHBG is a protein transporter of sex steroids that is synthesized primarily in the hepatocytes (25). Clinically, SHBG is usually detected with other sex hormone indicators to evaluate the bioactive estrogen/androgen levels of patients. Recently, increasing evidence from epidemiological studies has showed that lower SHBG levels are predictive of a higher risk for NAFLD and these studies highlighted SHBG as an important hepatokine in NAFLD progression (19, 20, 26). Saez-Lopez et al. found that SHBG overexpression could arrest the progression of NAFLD by regulating hepatic lipogenesis (27). Our previous clinical studies also confirmed the importance of SHBG in the diagnosis of NAFLD (28, 29).

In the current study, we found that SHBG alone had a comparable diagnostic accuracy as FLI index, with the AUC of 0.84, suggesting SHBG has high performance in predicting NAFLD. Thus, we used the multiple logistic regression model to screen out four independent variables, including SHBG and additional three parameters, one anthropometric index (BMI), one liver enzyme index (ALT/AST), and one lipids metabolism index (TG) respectively. The latter three parameters we screened are typical influencing factors for fatty liver and have been frequently used in predicting models. We believed that the variables selected should be more representative and efficient.

We then combined them to form a new formula SFI for predicting NAFLD. The AUROC of the SFI was 0.898 (95%CI, 0.865-0.926) and was significantly higher than that of FLI, LFS, LAP, and HSI (**Figure 3**). FLI, as a biochemical assessment model, can accurately identify ultrasonographic fatty liver and has been widely used in many cohorts of western and eastern countries (13, 30). Its high performance in predicting NAFLD was also confirmed in our previous study (28). In the current study, FLI still maintained good performance but was inferior to nomogram SFI in terms of AUC (0.84 vs. 0.89). Similarly, the remaining three models, HSI, LAP, and LSF were also inferior to SFI and could not do better than FLI. It indicates that the new model SFI is most likely to be a useful diagnostic test. Additionally, we also analyzed the ROCs in the subgroup of BMI< 23.0 kg/m^2^ to detect the diagnostic accuracy of the model in the non-obese NAFLD population. Predictably, the AUROC of the SFI in the non-obese people was 0.940 (95%CI 0.899-0.982), which presented a perfect diagnostic accuracy for NAFLD. It proves to us the feasibility of diagnosing non-obese NAFLD patients, and deserves of verification in larger populations.

In order to verify the validity of the predictive performance, we conducted the calibration curve and decision curve and demonstrated the higher performance and clinical utility of the nomogram in predicting NAFLD. The results suggest that nomogram SFI has a comparable function as abdominal ultrasound in diagnosing NAFLD.

The main strength of this study is that we developed a novel individualized prediction model for diagnosing NAFLD with the model contains key indicators that are easily available during routine examinations. To our knowledge, this is the first model that combines sex hormone related indicator and metabolic components to diagnose fatty liver. The model can be individually used as an easy-to-use screening tool to discriminate NAFLD and superior to previous non-invasive models and thus facilitate the management of patients.

We acknowledge that there are several limitations. First, in the retrospective single-center study, the enrolled subjects were mostly diabetes patients who had a higher incidence of metabolic disorders. Thus, our study may not be representative of the general Chinese population. However, it is gratifying that, through comparing SFI with other four prediction models, we can avoid the disadvantage of different sampled populations. Moreover, the AUROCs of the other four models were basically similar to the results reported in previous studies (14, 31, 32), which means that it may also be applicable to general populations, especially suitable for those with metabolic disorders. Second, due to the limitation of liver biopsy in general population, we served abdominal ultrasonography as the reference standard in building the diagnostic model of SFI. It is well known that ultrasonography tends to underestimate early hepatic steatosis. Therefore, liver biopsy can also be considered to verify the accuracy of the model in the future.

## Conclusion

Our study developed a novel clinical and laboratory-based nomogram, which innovatively incorporated an important predictor SHBG into the model. The nomogram has relatively good predictive ability in screening NAFLD in Chinese population. Clinicians can provide individualized plans to the subjects according to the risk assessment. High risk individuals should be referred for other diagnostic tests to confirm NAFLD, which will result in early lifestyle and medical interventions and prevent disease progression.

## Data availability statement

The raw data supporting the conclusions of this article will be made available by the authors, without undue reservation.

## Ethics statement

The studies involving human participants were reviewed and approved by Qilu Hospital (Qingdao), Shandong University. The patients/participants provided their written informed consent to participate in this study.

## Author contributions

YW and SZ contributed to the design and implementation of the research and was responsible for data curation and original draft preparation. HZ critically revised the manuscript for important intellectual content. GL and WY collected the samples. XH analyzed the data and drafted the manuscript. All authors contributed to the article and approved the submitted version.
