# Supplementary material for: SFI, a sex hormone binding globulin based nomogram for predicting non-alcoholic fatty liver disease in the Chinese population
Source: Front Endocrinol (Lausanne). 2023 Jun 6;14:1176019. doi: 10.3389/fendo.2023.1176019 (PMC10276183; doi:10.3389/fendo.2023.1176019)
Supplement: Supplementary file 1 [file Table_1.docx]

**Supplement Table S1. Baseline characteristics of patients with and without NAFLD in the validation dataset.**

|  | Total  (n=414) | non-NAFLD  (n=231) | NAFLD  (n=183) | *P* value |
| --- | --- | --- | --- | --- |
| Sex (Male/Female) | 257/157 | 127/104 | 130/53 | 0.001 |
| Age (years) | 61.14 ± 14.05 | 64.68 ± 12.52 | 56.67 ± 14.63 | < 0.001 |
| Mets (%) | 245 (59.2) | 107 (46.3) | 138 (75.4) | < 0.001 |
| Smoking (%) | 93 (22.5) | 39 (16.9) | 54 (29.5) | 0.002 |
| Drinking (%≥1 /w) | 45 (10.9) | 13 (5.6) | 32 (17.5) | 0.000 |
| SBP (mmHg) | 134.48 ± 19.17 | 133.58 ± 18.44 | 135.61 ± 20.04 | 0.286 |
| DBP (mmHg) | 77.10 ± 12.77 | 74.74 ± 12.65 | 80.07 ± 12.32 | < 0.001 |
| BMI (kg/m^2^) | 24.99 ± 3.47 | 23.71 ± 2.77 | 26.61± 3.59 | < 0.001 |
| WC (cm) | 91.64 ± 10.31 | 88.18 ± 9.27 | 96.01 ± 9.89 | < 0.001 |
| ALT (U/L) * | 26.07 ± 18.69 | 19.73 ± 10.89 | 34.07 ± 22.97 | < 0.001 |
| AST (U/L)* | 21.841 ± 10.79 | 20.01 ± 8.72 | 24.15 ± 12.60 | < 0.001 |
| ALT/AST | 1.16 ± 0.46 | 0.99 ± 0.38 | 1.38 ± 0.49 | < 0.001 |
| GGT (U/L)* | 33.42± 28.69 | 24.82 ± 20.34 | 42.39 ± 34.69 | < 0.001 |
| TC (mmol/L) | 4.53 ± 1.15 | 4.33 ± 1.05 | 4.50 ± 1.16 | 0.059 |
| TG (mmol/L)* | 1.97 ± 1.90 | 1.35 ± 1.00 | 2.37 ± 1.18 | < 0.001 |
| HDL-C (mmol/L) | 1.12 ± 0.46 | 1.21 ± 0.42 | 1.02 ± 0.48 | < 0.001 |
| LDL-C (mmol/L) | 2.40 ± 0.86 | 2.32 ± 0.81 | 2.34 ± 0.84 | 0.382 |
| FPG (mmol/L) | 7.99 ± 3.83 | 7.50 ± 3.74 | 8.61 ± 3.86 | 0.003 |
| HbA1c (%) | 8.61 ± 2.37 | 8.52 ± 2.55 | 8.71 ± 2.12 | 0.414 |
| FINS (mU/L)* | 10.77 ± 16.00 | 8.18 ±8.03 | 13.71 ± 21.42 | < 0.001 |
| FCP (pmol/L)* | 764.10 ± 437.54 | 619.01 ± 381.96 | 932.04± 438.73 | < 0.001 |
| HOMA-IR | 3.85 ± 1.95 | 3.23 ± 1.26 | 4.57 ± 2.33 | < 0.001 |
| SHBG (nmol/L) | 35.79 ± 19.35 | 45.22 ± 19.46 | 23.87 ± 10.77 | < 0.001 |

Continuous data were performed as the Student’s T-test or Mann-Whitney’s U-test* and presented as means **±** SD. Categorical variables are presented as proportion and performed as χ^2^**-**test.
