# Supplementary figures and images for: SFI, a sex hormone binding globulin based nomogram for predicting non-alcoholic fatty liver disease in the Chinese population
Source: Front Endocrinol (Lausanne). 2023 Jun 6;14:1176019. doi: 10.3389/fendo.2023.1176019 (PMC10276183; doi:10.3389/fendo.2023.1176019)

**Supplement Figure S1. ROC curves for predicting NAFLD in patients of BMI<23kg/m2.**


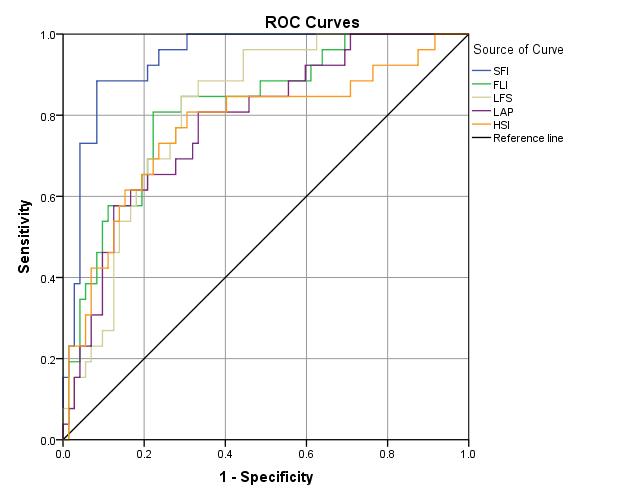

Supplement: Supplementary file 2 [file DataSheet_1.docx]
